# The Brave Patient after 80—Satisfaction with Visit and Individual Determinants of Proactive Patient Attitude among the Oldest General Practice Users

**DOI:** 10.3390/ijerph19106214

**Published:** 2022-05-20

**Authors:** Marta Rzadkiewicz, Mariusz Jaworski, Dorota Włodarczyk

**Affiliations:** 1 Department of Health Psychology, Medical University of Warsaw, 01-575 Warsaw, Poland; dorota.wlodarczyk@wum.edu.pl; 2 Department of Education and Research in Health Sciences, Medical University of Warsaw, 01-575 Warsaw, Poland; mariusz.jaworski@wum.edu.pl

**Keywords:** community dwelling, primary care, patient activation, patient satisfaction, self-efficacy, older adults, age-friendly, patient-centered

## Abstract

Background. A patient’s adherence to a course of treatment depends on the individual’s activation, the quality of patient–clinician relations, attitudes, self-efficacy, or positive emotions. Patient proactive attitude (PAA) is seldom researched among the oldest healthcare users. This study was designed to identify predictors of PAA toward health and treatment among community-dwelling general practice patients aged 80+, and was based on a PRACTA (PRomoting ACTive Aging) project. Methods. Patients (*n* = 658), aged 80+ visiting a general practitioner (GP) filled in the PRACTA attitude toward treatment and health scale and the PRACTA self-efficacy scale questionnaires. Sociodemographic factors, self-reported health status, and satisfaction with the visit were analyzed as independent factors. Results. Attitudes toward treatment and health scores were predicted by marital status, living alone or not alone, hospitalization the prior year, level of impairment, and satisfaction with visit. However, some differences were observed depending on the device’s subscale. Self-efficacy score was determined by marital status, living alone or not alone, prior hospitalization, and satisfaction with visit. We did not find an effect of age or gender on PAA. Patient satisfaction with visit was the strongest predictor of all PAA dimensions. Conclusion. Higher visit satisfaction helps to retain a PAA among seniors 80+. Screening questions about living situation, marital and functional status, emotional state, and recent history of hospitalization might help GPs additionally anticipate PAA level and adjust their actions accordingly.

## 1. Introduction

The effectiveness of healthcare depends on patient proactive attitude (ProActive Attitude—PAA) toward treatment and health, as attitude impacts patient engagement, empowerment and self-management [1,2,3]. As all of these are strongly associated with active participation in treatment, they are increasingly seen as essential aspects of medical care for all ages [2,3,4,5]. Yet these factors are often assigned overlapping and interrelated definitions. Patient engagement, for example, is consistently perceived as both a core pillar [6] and a core challenge [7] in improving healthcare systems for older people. Still, it has been defined in many ways, ranging from patient involvement in self-care and care-related decisions [8,9] to patient participation in research design and interpretation [6].

A patient’s attitude is recognized as proactive when the patient perceives his or herself as thoroughly understanding their condition, confident, having a positive approach, and being able to maintain the recommended postvisit self-management [10,11,12,13]. Studies exploring a range of health conditions and patient groups have revealed that patients’ activation in general improves their healthcare experience and health outcomes [14,15]. Kinney and co-workers have shown that patients with lower levels of activation, in contrast, are at greater risk of hospital or emergency department use [16].

Many older adults take a less active approach to their own health. Self-efficacy decreases with age, while the locus of control concerning powerful others (usually doctors) and patient helplessness regarding health self-management increase [17]. Nevertheless, a growing body of literature shows that patient activation level can be changed through intervention [13,18], even among more mature patients [19]. Therefore, we must identify the determinants of a proactive attitude toward health to promote it in the aim of improving healthcare outcomes and reducing costs for the senior population.

Several studies have already identified some determinants of PAA. First, PAA seems to vary markedly between individuals and between patient groups [20,21]. Second, the following overlapping groups of determinants have been identified:

- Sociodemographic factors such as age, marital status, employment, education, or income [21,22,23];
- health-related factors such as BMI, disease severity, comorbidities [22], and self-reported health or illness-related burden [24];
- care-related factors such as provider access, time spent with provider [21], and satisfaction with care and healthcare use [25];
- psychological factors such as illness perception, anxiety [22,23], health literacy [25] and fear of recurrence [21].

Among the factors related to PAA, self-efficacy seems to be particularly well-researched. For older people, self-efficacy is diminished by depression [26], anxiety [27], frailty [26], experience of discrimination [28], and functional decline [29]; and promoted by perceived social support [30].

Yet the factors affecting PAA have been studied mostly in specific chronic illnesses or clinical settings. Far less is known about what influences PAA in relatively well-functioning, community-dwelling older people. Knowing the predictors of PAA in this population will help us develop interventions reducing the present deficiencies and promote patient engagement.

The present work unifies classical understanding of the concepts of attitude and self-efficacy in relation to health with an activity-based approach. The aim of this study was to identify predictors of PAA among community-dwelling general practice users aged 80+. The following research questions guided the analysis:

1. Is PAA among patients aged 80+ related to visit satisfaction?
2. Which self-reported measures of health status can predict PAA among seniors postvisit?
3. What are the sociodemographic determinants of PAA for seniors aged 80+?

## 2. Materials and Methods

### 2.1. The Research Project

The data was extracted from the PRACTA—*activating the elderly in medical practice* project, described elsewhere in detail [10]. The PRACTA (PRomoting ACTive Aging) study was developed to prospectively analyze GPs’ competences in communication with and activation of mature patients, along with exploring patients’ experiences directly. Patients aged 50 or more were examined both before and after visiting a GP in their practice. We have chosen the cut-off point of 50 years old to be consistent with the Survey of Health, Ageing, and Retirement in Europe (SHARE) which is representative of the noninstitutionalized population aged 50 years or older in 15 participating countries, including Poland.

The data was collected in 151 general practices in central Poland, from 491 GPs (mean age M = 49.3 years, range 26–86, 64% women, response rate 50%). Doctors had on average 23 years of professional experience, 91% had a specialty in family or/and internal medicine. The majority of GPs (54%) perceived the rate of their senior patients (aged 65 years or more) as 50% or higher, yet only 48% had any formal education in geriatrics (a course or seminar at least once in their carrier). The GPs were contacted twice—before and after a dedicated educational intervention. Accordingly, two independent groups of their patients were approached. All measures underwent content validation through an expert panel including health psychologists and nurses, and a pilot study followed by factorial analysis and reliability tests. The psychometric tools were used in Polish; however, a translation into English is available for informative and reporting purposes at www.practa.wum.edu.pl (accessed on 19 April 2022). This research project was approved by the Bioethics Committee of the Medical University of Warsaw (KB/10/2014).

### 2.2. Participants

Participants of the present study were selected from the first group of patients, i.e., approached before implementation of the intervention for GPs. This was a convenience sample (*n* = 4921, with response rate of 75.5%, age range 50–98, mean age 68.9 years, 57.7% women). The current sample consists of 658 older adults, aged ≥80 years (671 patients were eligible, but satisfactory data was unavailable for 13; eligibility rate 98.06%). As for the age threshold, we have chosen 80 years old following the literature review. The 80+ age bracket is a frequent choice for selecting very old persons with the prevalence of functional limitations growing exponentially.

Included were patients aged 80+ awaiting a scheduled appointment with a GP participating in the PRACTA study who were capable of independently answering our questionnaires (help filling in the fields was accepted) and who gave written consent. Each participant received all relevant information about the study’s purpose and procedures.

### 2.3. Independent Variables

Directly before the GP appointment, each patient reported basic sociodemographic data (gender, age, marital status, financial status, living situation (alone/not alone)) and health-related data (self-rated health, the health impact on activities scale, hospitalization during the last year, number of diagnosed diseases). Health-related limitations on everyday activities were assessed with the Health Impact on Activities scale (10 items), which evaluates efficacy among independent community-dwelling older adults and was described earlier [10]. Participants rated how much their health status limited their functioning (e.g., their ability to do their own shopping) on a four-point Likert scale (from “does not limit at all” to “limits very much”). The score was interpreted as reflecting low, moderate, or high impairment level based on the mean score and standard deviation (mean = 2.10; SD = 0.851).

After the appointment, each participant filled out the questionnaires for the Patient Satisfaction with Visit Scale (PSVS), PRACTA Attitude toward Treatment and Health scale (PRACTA-ATH), and PRACTA self-efficacy scale (PRACTA-self-efficacy). The PSVS consisted of seven items such as: Would you like to come to this doctor again? Patients responded on a seven-point Likert scale, from definitely no to definitely yes. In the present sample, this tool yielded a reliability coefficient (Cronbach’s α) of 0.956.

### 2.4. Outcome Variables

Assessment of PAA as outcome was based on the PRACTA-ATH and PRACTA-self-efficacy scores as described previously [10,31]. The PRACTA-ATH consists of four subscales (cognitive, positive emotions, negative emotions, motivation; 16 items; Cronbach’s α = 0.873). The unidimensional PRACTA-self-efficacy consists of three questions (Cronbach’s α = 0.919). Each item began with the expression: “As a result of this visit at the doctor …” and was followed by a dimension’s specific ending, e.g., “I’m going to participate in the treatment actively” for the dimension of motivation section, or “I think I can influence how I’ll feel in the future” for self-efficacy. The answers were registered on the seven-point Likert scale from definitely no to definitely yes. Higher scores (excepting the ATH negative emotions subscale) indicate a more PAA. Assuming that PAA is a complex construct, we analyzed in parallel the individual domains of PAA in detail.

### 2.5. Analysis

The data were analyzed with IBM SPSS 24 software (IBM, Armonk, NY, USA). Mean (M) and SD were calculated for all variables. To identify the predictors of outcome variables (patients’ ATH and self-efficacy scores), we used a generalized linear model (GENLIN, distribution Gamma, link identity), which is based on regression analysis, but is applicable to non-normal data distributions [32,33].

## 3. Results

Participants’ mean age was 83 years (standard deviation—SD = 3.26, range 80–96). The majority of patients were female (62%), about 1/3 were married (the majority were widowed), and 1/3 were living alone. About 1/3 declared one or no diseases. Self-rated health had a mean value close to the middle point in the scale, described as *average* (M = 3.19, SD = 0.79, range 1 to 5). About 6 out of 7 patients had been hospitalized within the last year (including emergency room visits or one-day observation). More detailed descriptive statistics of input variables are presented in Table 1.

Both the PRACTA-ATH and PRACTA-self-efficacy results were skewed toward higher scores, with most scores falling above the central value (means 5.22–5.46), with the exception of negative emotions (M = 3.21, SD = 1.56). The results of our GENLIN analysis identifying predictors of PAA on dimensional PRACTA-ATH scores along with PRACTA-self-efficacy score are presented in Table 2.

Cognitive ATH was predicted by marital status, living situation, hospitalization in the last year, number of diseases, and PSVS score. Patients perceived their knowledge about their treatment as more adequate when they were: not widowed, living alone, hospitalized in the last year, diagnosed with fewer illnesses (although no significant particular pairwise comparison was observed, score for knowledge about treatment gradually decreased as the number of illnesses increased), and scoring higher on the PSVS. Participants scored higher on the positive emotions subscale of the PRACTA-ATH when they were: not widowed, hospitalized in the last year, and less impaired. A positive relationship was found between the positive emotions subscale of the PRACTA-ATH and PSVS. The negative emotions subscale of the ATH was mainly governed by patient satisfaction with a small additional effect of financial situation. Lower PSVS score and better financial situation were associated with more negative emotions. The motivational aspect of ATH was predicted by marital status, living situation, and the health impact on activities score: those who were widowers, living with others, not hospitalized in the last year, and moderately impaired were less motivated; yet the strongest predictor of higher motivation was greater patient satisfaction. Interestingly, gender, age, and self-rated health were not related to any ATH dimension.

Like ATH, perceived health-related self-efficacy (PRACTA-self-efficacy) was higher for participants who were not widowers, lived alone, and had been hospitalized in the last year. Noteworthy PSVS had a strong boosting effect on self-efficacy. In general, satisfaction score was related to each outcome variable.

## 4. Discussion

To sum up, patient satisfaction with a visit was more strongly associated with all outcome measures than any other factor introduced into the GENLIN statistical model by an order of magnitude. This result answers the first research question of our study. To answer the second research question, hospitalization in the prior year and the health impact on activities score were found to be the main predictors of PAA among self-reported measures of health status. Number of diseases and self-rated health had marginal or no effect in the studied population. Additionally, patients’ marital status and whether they lived alone or not alone were significantly associated with various dimensions of PAA, while gender or age had no meaning, which answers the third research question.

Our group of participants is in some aspects similar to representative senior samples from national studies, while in other characteristics it differs substantially. According to Statistics Poland (stat.gov.pl/en, accessed on 12 April 2022), 70% of citizens aged 80 or more are women, thus representing a slightly higher rate than in present research. Of this age group, 38% sees their health status as average (close to the mean in our sample), and 25% has been hospitalized in the last year (here 86.5%). The same source informs us that in the age group of 75+ years old, 25% declare strong problems with daily activities (16% in this study), and 79% have long-lasting health problems (70.5% of PRACTA 80+ participants have at least two diagnosed problems). The hospitalization rate shows the greatest discrepancy with the representative sample of Poles; however, the inclusion criteria and the understanding of hospitalization were different in these datasets.

Factors referring to patients’ individual situations: being widowed, living with others, not being hospitalized in the last year, and having a higher degree of health-related impairment in everyday life might be linked to lower levels of health-related activity in old age. On one hand, these findings confirm earlier reports about the particular vulnerability of the bereaved elderly [34]. On the other hand, the positive effects of living alone and having recently been hospitalized are intriguing and may be related to motivation. Maintaining the maximum independence and the ability to stay in the community after being hospitalized at the age of 80+ can act as either a positive or negative reinforcement for self-care, thus inducing PAA. A positive reinforcement may be, for example, triggering the desire to maintain the current quality of life for as long as possible, while a negative one can refer to efforts to avoid further hospitalization or general health deterioration. Moreover, the PAA-boosting effect of hospitalization can result from several other reasons: outcomes of hospital treatment may contribute to noticeable health improvement in this group, or hospitals may be more supportive for those patients than other health services. As the proportions of hospitalized and non-hospitalized participants in our study were unequal, the above suppositions are only hypothetical. Complementing this context, it is worth noting that patient life satisfaction and more limitations on daily living have previously been related to more active participation in GP visits [35].

The present study revealed that, in participants aged 80+, gender and age were not related to PAA, and financial situation, number of diseases, and self-rated health status played negligible roles. This is not in line with previous studies [36] or with common beliefs resulting from the medical approach to aging [37]. After comparison with younger participants of the PRACTA project (50–79 years old, *n* = 4209), participants aged 80+ received similar scores on the cognitive, positive emotions, and motivation subscales of the PRACTA-ATH (Kolmogorov–Smirnov’s Z = 1.07, Z = 0.78, and Z = 0.66, respectively, all *p* > 0.05), but higher scores were noted on the negative emotions subscale (Z = 1.76, *p* < 0.005) and slightly lower scores on PRACTA-self-efficacy scales (Z = 1.55, *p* = 0.016). Although no effect of age was observed within the present study group, the above difference shows that the eldest GP users can present with more negative emotions and lower levels of self-efficacy than patients aged 50–79 [17]. Further studies should assess the possibility of interactions between the predictors, which to date have been analyzed separately.

Visit satisfaction was the factor most strongly linked to all assessed dimensions of PAA among seniors of 80+. Although the definition of patient satisfaction is still “under construction” [38,39,40], a growing body of evidence, including this study, suggests that patient satisfaction influences patient engagement, self-care, and adherence [36].

Older patients’ satisfaction with medical care is usually high, especially considering the formal aspects of the doctor–patient relationship (e.g., politeness). Yet there remains room for improvement: individual approach, recognition of patients’ personal situation, and shared decision-making can enhance visit satisfaction among this population [41,42]. Improvements in patient–provider communication and awareness of mental health resources are also determinants of visit satisfaction in general practice [43]. It is worth noting that these factors might also promote practices aimed at reducing negative emotions and/or enhancing self-efficacy [44]; for example, negative emotions (doubt, fear) can be more specifically addressed during appointments. The tool we used to measure patient satisfaction also evaluates the GP’s perceived response to the patient’s visit-related expectations, whether the patient felt that the GP gave them enough time, and the patient’s willingness to choose or recommend this GP in the future. These aspects were very sensitive predictors of all of our outcomes. As the motivational dimension of the ATH theoretically represents a more advanced stage of attitude activation, we must acknowledge all of its numerous determinants.

### 4.1. Strengths and Limitations

Some overlap presumably exists between self-reporting of PAA and self-reporting of visit satisfaction. Moreover, PAA and PSVS yield subjective scores, both of which might be influenced by personality traits such as optimism or locus of control. Even so, patients’ rating of their own health, which was also subjective, was only weakly linked with general PRACTA-ATH score (with a Wald’s χ² value > 60 times weaker than that for ATH and satisfaction). Community-dwelling seniors probably represent a healthier, more independent and non-institutionalized fraction of all the seniors served by GPs. Additionally, they were patients of GPs who were willing to participate, from facilities which agreed to be a part of the project. These facts pose the problem of highly possible selection bias; therefore, any conclusions from this study should be generalized to the entire age group with care.

The added value of this study is confirmation that among the oldest patients in general practice, satisfaction with the visit is the strongest correlate of patients’ activation in terms of their knowledge, motivation and self-efficacy. It is also related to a better ratio of positive and negative emotions. Gender, further age increase, the degree of multiple diseases, and accompanying subjective health evaluation were not related to proactive attitude in seniors of 80+. This may show the specificity of this group and contributes to clearing up misconceptions about the oldest patients. These results indicate the need to monitor the factors contributing to satisfaction with the visit, including the doctor–patient relationship and those outside the doctors’ offices.

### 4.2. Implications for Practice, Policymakers, and Research

To promote proactive attitudes among the oldest patients in general practice, special attention should be paid during appointments to negative emotions such as fear and doubt, as well as their perception of their own self-efficacy. Additional attention to these issues is needed for patients who are widowed, greatly impaired, not recently hospitalized, and/or not living alone. Understanding the significance of patient satisfaction is necessary in any study of the effects of patient PAA on treatment and health. Further exploration should address the possibility of causality in our observed associations.

The mediating/moderating effect of patient satisfaction on PAA and treatment outcomes and the role of the patient–doctor relationship are also worth clarifying. The possibility of a motivating effect on PAA resulting from living alone or having been hospitalized in the last year warrants further investigation.

## 5. Conclusions

Among the oldest general practice users, higher visit satisfaction helps them retain a PAA toward treatment and health on all measured dimensions. Moreover, visit satisfaction is much more strongly related to PAA than any other individual characteristic. Therefore, improving visit satisfaction by strengthening physicians’ communication skills is crucial. Screening questions about living situation, marital status, emotional state and recent history of hospitalization might help GPs to anticipate whether additional steps are necessary to ensure PAA.

This study identifies predictors of proactive attitude towards health and treatment among community-dwelling adults aged 80 and over. Its implications might inspire individual physicians, health management organizations, policy makers, or public health professionals to provide tailored, age-friendly medical care. The results could also help to reduce the perception of the oldest GP patients as passive healthcare recipients.

## Figures and Tables

**Table 1:** Descriptive statistics for independent variables.

Patient Characteristics (*n* = 658)	Mean (SD) or Percentage	Range, Scale, or Other Metrics
Gender	62.2% female	
Age, M (SD)	83.33 (3.27)	Range 80–96
Marital status	57.9% widowed 33.4% married	
Living situation	33.3% living alone	Alone or not alone
Financial situation, M (SD)	2.93 (0.83)	1—poor to 5—good
Hospitalized in the last year (yes/no)	13.5% no	Including visits to the Emergency Department and one-day observation
Number of diseases	29.1% none or one 28.6% four or more	
Health impact on activities score	26.4% low 16.0% high	10 items, scored as low, medium, or high
Self-rated health status, M (SD)	3.19 (0.79)	1—very good to 5 very poor
PSVS, M (SD)	5.49 (1.04)	7 items, range 1–7

**Table 2:** Factors predicting patient proactive attitude (PAA) on separate dimensions and general PRACTA-ATH score plus the self-efficacy scale score (Wald χ² value, *p* value). Shadowed are all nonsignificant effects, with detailed values of those with *p* < 0.2.

PAA	ATH Cognitive	ATH Positive Emotions	ATH Negative Emotions	ATH Motivation	Self-Efficacy
Wald χ² for the model (*p*)	462.10	341.273	66.695	223.534	425.400
	(*p* < 0.001)	(*p* < 0.001)	(*p* < 0.001)	(*p* < 0.001)	(*p* < 0.001)
Gender	0.517	0.521	0.162	0.015	2.371
	*p* > 0.05	*p* > 0.05	*p* > 0.05	*p* > 0.05	*p* = 0.124
Age	1.016	0.668	0.211	0.003	0.000
	*p* > 0.05	*p* > 0.05	*p* > 0.05	*p* > 0.05	*p* > 0.05
Marital status	22.481	20.398	3.964	15.084	13.050
	*p* < 0.001	*p* < 0.001	*p* > 0.05	*p* = 0.002	*p* = 0.005
Living situation	19.223	3.601	1.714	13.961	9.123
	*p* < 0.001	*p* = 0.058	*p* = 0.190	*p* < 0.001	*p* = 0.003
Financial situation	0.649	0.353	3.953	1.964	0.081
	*p* > 0.05	*p* > 0.05	*p* = 0.047	*p* = 0.161	*p* > 0.05
Hospitalized in the last year	22.312	13.244	2.336	5.606	23.558
	*p* < 0.001	*p* < 0.001	*p* = 0.126	*p* = 0.018	*p* < 0.001
Number of diseases	8.249	7.528	2.410	2.868	4.984
	*p* = 0.041	*p* = 0.057	*p* > 0.05	*p* > 0.05	*p* = 0.173
HIA	2.051	16.060	3.618	13.452	3.043
	*p* >0.05	*p* < 0.001	*p* = 0.164	*p* = 0.001	*p* >0.05
Self-rated health status	1.657	0.138	1.881	1.939	1.724
	*p* = 0.198	*p* > 0.05	*p* = 0.170	*p* = 0.164	*p* = 0.189
PSVS	291.375	164.796	10.653	77.747	209.100
	*p* < 0.001	*p* < 0.001	*p* = 0.001	*p* < 0.001	*p* < 0.001
	β = 0.627	β = 0.614	β = −0.272	β = 0.434	β = 0.701
	CI 0.595 to 0.750	CI 0.520 to 0.707	CI −0.435 to −0.109	CI 0.338 to 0.531	CI 0.606 to 0.796
	ATH cognitive	ATH positive emotions	ATH negative emotions	ATH motivation	Self-efficacy

ATH—attitude toward treatment and health; HIA—health impact on activities (3 levels); PSVS—patient satisfaction with visit scale.

## Data Availability

The datasets generated and analyzed during the current study are available from the corresponding author on reasonable request.
